# Supplementary material for: Interpreting population and family-based genome-wide association studies in the presence of confounding
Source: bioRxiv. 2023 Feb 27:2023.02.26.530052. Preprint. [Version 1] doi: 10.1101/2023.02.26.530052 (PMC10002712; doi:10.1101/2023.02.26.530052)
Supplement: Supplement 1 [file NIHPP2023.02.26.530052v1-supplement-1.pdf]

bioRxiv preprint doi: https://doi.org/10.1101/2023.02.26.530052; this version posted February 27, 2023. The copyright holder for this preprint (which was not certified by peer review) is the author/funder, who has granted bioRxiv a license to display the preprint in perpetuity. It is made available under aCC-BY 4.0 International license.

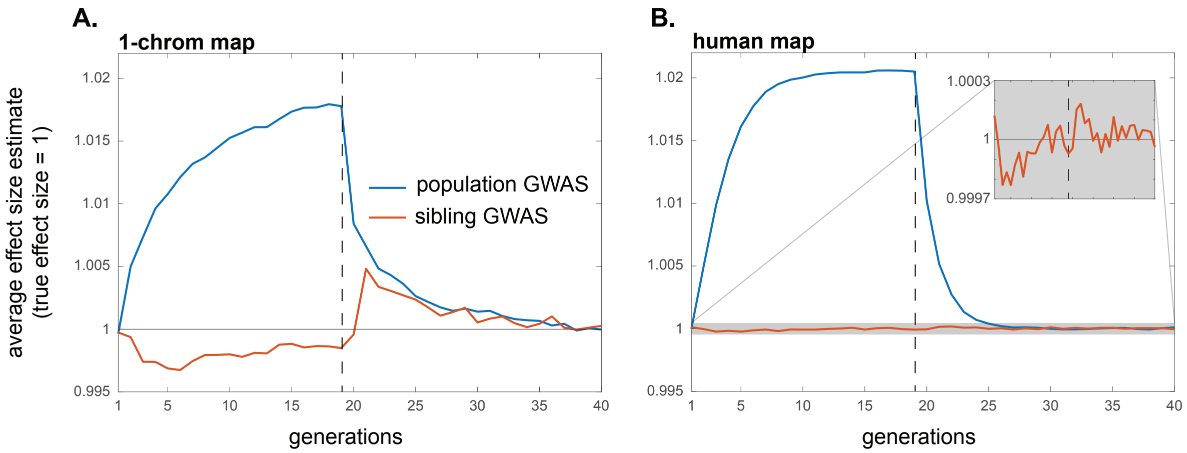

Figure S1: Cross-trait assortative mating influences effect size estimates at loci that affect the study trait, although this influence is second-order relative to that on effect size estimates at loci that do not affect the study trait but do affect the other trait involved in assortative mating (note the scale of the y-axis). Simulations are the same as in Fig. 2.
